# Supplementary material for: Identification of photoperiod-induced specific miRNAs in the adrenal glands of Sunite sheep (Ovis aries)
Source: Front Vet Sci. 2022 Jul 22;9:888207. doi: 10.3389/fvets.2022.888207 (PMC9354845; doi:10.3389/fvets.2022.888207)
Supplement: Supplementary file 1 [file Data_Sheet_1.ZIP › Supplementary file/miRDeep2 file.zip!/miRDeep2 file/Novel_Structure/SP42A2/Novel_640.pdf]

Provisional ID	:	NC_019470.2_26437
Score total	:	2
Score for star read(s)	:	-1.3
Score for read counts	:	0
Score for mfe	:	1.7
Score for randfold	:	1.6
Score for cons. seed	:	
Total read count	:	20
Mature read count	:	18
Loop read count	:	0
Star read count	:	2

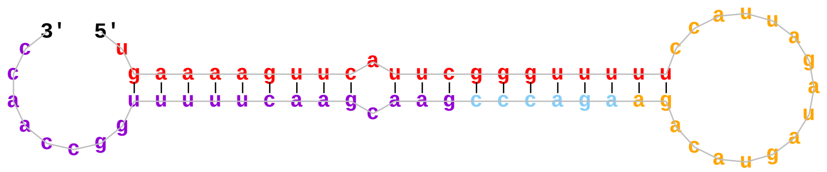

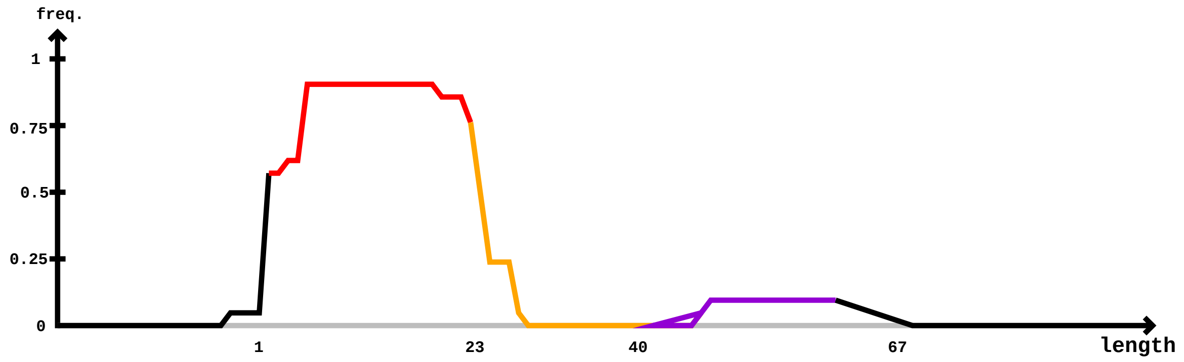

Mature **Star**

```
5'- cugccuucuauugggcuggcugaaaaguucauucggguuuuuccauuagauaguacagaagacccgaacgaacuuuuuggccaaccaauacauuuuccaucucuguucuucc  -3'   obs
    cugccuucuauugggcuggcugaaaaguucauucggguuuuuccauuagauaguacagaagacccgaacgaacuuuuuggccaaccaauacauuuuccaucucuguucuucc        exp
    ........(((((..((((((((((((((.(((((((((((..............))))))))))).)))))))))))))).))))).........................  reads   mm   sample
    ................uggcugaaaaguucauuUgggu..........................................................................  1       1    seq
    ....................ugaaaaguucauucggguuuu.......................................................................  2       0    seq
    ....................ugaaaaguucauucggguuuuu......................................................................  9       0    seq
    ......................aaaaguucauuUggguuuuuc.....................................................................  1       1    seq
    ........................aaguucauucggguuuuuc.....................................................................  1       0    seq
    ........................aaguucauucggguuuuuccau..................................................................  4       0    seq
    ........................aaguucauucggguuuuuccauu.................................................................  1       0    seq
    .................................................................gaacgaacuuuuuggccaacU..........................  1       1    seq
    ..................................................................aacgaacuuuuuggccaaU...........................  1       1    seq
```
